# The “Cerebrospinal Fluid Sink Therapeutic Strategy” in Alzheimer’s Disease—From Theory to Design of Applied Systems

**DOI:** 10.3390/biomedicines10071509

**Published:** 2022-06-25

**Authors:** Thomas Gabriel Schreiner, Manuel Menéndez-González, Bogdan Ovidiu Popescu

**Affiliations:** 1 Faculty of Medicine, University of Medicine and Pharmacy “Carol Davila”, 050474 Bucharest, Romania; bogdan.popescu@umfcd.ro; 2 Department of Neurology, University of Medicine and Pharmacy “Gr. T. Popa”, 700115 Iasi, Romania; 3 Department of Electrical Measurements and Materials, Faculty of Electrical Engineering and Information Technology, Gheorghe Asachi Technical University of Iasi, 700050 Iasi, Romania; 4 Department of Medicine, University of Oviedo, 33006 Oviedo, Spain; manuelmenendez@gmail.com; 5 Department of Neurology, Hospital Universitario Central de Asturias, 33006 Oviedo, Spain; 6 Instituto de Investigación Sanitaria del Principado de Asturias, 33006 Oviedo, Spain; 7 Neurology Department, Colentina Clinical Hospital, 020125 Bucharest, Romania; 8 Laboratory of Cell Biology, Neurosciences and Experimental Myology, ‘Victor Babes’ National Institute of Pathology, 050096 Bucharest, Romania

**Keywords:** Alzheimer’s disease, amyloid-beta, cerebrospinal fluid, therapy, implantable device, clearance

## Abstract

Alzheimer’s disease (AD) is a global health problem, with incidence and prevalence considered to increase during the next decades. However, no currently available effective treatment exists despite numerous clinical trials in progress. Moreover, although many hypotheses are accepted regarding the pathophysiological mechanisms of AD onset and evolution, there are still many unknowns about the disorder. A relatively new approach, based on the amyloid-beta dynamics among different biological compartments, is currently intensely discussed, as it seems to offer a promising solution with significant therapeutic impact. Known as the “cerebrospinal-fluid-sink therapeutic strategy”, part of the “three-sink therapeutic strategy”, this theoretical model focuses on the dynamics of amyloid-beta among the three main liquid compartments of the human body, namely blood, cerebrospinal fluid, and the (brain) interstitial fluid. In this context, this article aims to describe in detail the abovementioned hypothesis, by reviewing in the first part the most relevant anatomical and physiological aspects of amyloid-beta dynamics. Subsequently, explored therapeutic strategies based on the clearance of amyloid-beta from the cerebrospinal fluid level are presented, additionally highlighting their limitations. Finally, the originality and novelty of this work rely on the research experience of the authors, who focus on implantable devices and their utility in AD treatment.

## 1. Introduction

A huge and heterogeneous amount of research has been conducted in recent years in the search for an efficient therapeutic approach to neurodegenerative diseases [1,2,3]. Most clinical trials focused on the finding of a potent therapy for Alzheimer’s disease (AD), as this disorder is the most common type of dementia [4]. However, the majority ended with disappointing results. AD is currently a great burden for individuals and for the society, from both the healthcare and socioeconomic point of view [5]. According to the latest statistical data, AD is affecting at present more than six million Americans aged 65 and older [6] and is associated with increased mortality rates [7]. Moreover, the predictions are unfavorable, with AD incidence and prevalence expected to increase in the next decades [8]. In this context, despite previous failed studies, all data from the past should be reconsidered and integrated in an innovative manner in future research on AD therapies.

First described more than 100 years ago by Alois Alzheimer [9], AD is still incompletely understood, with the etiology and the entire pathophysiological mechanism yet to be determined [10]. Before even starting to consider another theoretical approach, the return and rethinking of already-established hypotheses are mandatory, as they have still many relevant data to deliver. Many theories exist and are able to explain, at least partially, the onset and evolution of AD, and among the most discussed and accepted, worth to be mentioned are the cholinergic hypothesis [11], the theories related to the impact of neuroinflammation as the main trigger for subsequent neurodegeneration [12], the misfolded Tau protein hypothesis [13], and the role of free radicals and reactive oxygen species in neuronal damage [14]. Of great importance remains the amyloidogenic hypothesis [15], as pathological amyloid-beta (Aβ) aggregation in early soluble oligomers and subsequent insoluble amyloid plaques represent the central hallmark for AD [16]. Furthermore, all other cellular and molecular dysregulations are connected via explained or still unknown mechanisms to the amyloidogenic pathway and the pathological formation of senile plaques [17]. The focus on Aβ, unraveling its role in the context of the complex and intricate neurodegenerative mechanisms, is essential for a better understanding of AD and for the development of effective therapies.

The therapeutic modalities in AD are characterized by a vast heterogeneity of explored methods. The majority have, however, proved to be unsuccessful in most clinical trials [18]. One major limitation derives from the fact that some therapeutic interventions focus only on one aspect of the disease, such as limiting the neuroinflammation [19] or modulating the cholinergic system [20]. Indeed, anticholinergic drugs such as Donepezil and Galantamine are among the first approved and still in-use medications in the daily clinical practice [21]. Memantine, an N-methyl-D-aspartate (NMDA) receptor blocker, one of the most frequently prescribed medications in the United States, is administered alone or in combination therapy in moderate-to-severe AD patients [22]. Very recently, the first Food and Drug Administration (FDA)-approved Aβ-directed monoclonal antibody was introduced as a therapeutic option for AD patients [23], with data from the open-world still to be published.

A promising therapeutic area is related to methods designed to enhance Aβ clearance. Given the presence of Aβ in different biological compartments of the human body, including the peripheral circulation, the incipient therapeutic strategies are focused on reducing the Aβ from the blood, hoping to achieve the final goal of reducing the cerebral Aβ level. Several procedures were tested, with the most relevant ones being the neprilysin-based direct cleavage [24], passive and active immune-based approaches [25], and direct elimination via plasmapheresis [26]. Despite no significant clinical improvement [27], the reduction of AD-associated peripheral biomarkers suggests the validity of these methods and encourages their combined use at the central nervous system (CNS) level.

In the context of growing evidence related to AD pathogenesis and therapeutic modalities, the main aim of this work is to focus on a relatively recent theoretical model which gathers prior knowledge from different hypotheses, mainly the amyloidogenic pathological pathway. Known as the “three-sink therapeutic strategy”, this model suggests that there exists a dynamic equilibrium of Aβ among the three most relevant biological compartments, namely blood, cerebrospinal fluid (CSF), and (brain) interstitial fluid (ISF). Subsequent to the detailed presentation of the anatomical and physiological aspects of this theoretical paradigm, the most important AD therapies targeting Aβ are reviewed, with a focus on methods enhancing Aβ clearance. In the final part, the opportunity of introducing implantable devices into the bedside management of AD patients is discussed, with the authors presenting, in a detailed manner, their practical experience with intrathecal CSF pumps.

## 2. Revising the Aβ Hypothesis and Dynamics—From the Anatomical−Physiological to Pathological Aspects

### 2.1. Aβ Formation and Dynamics in Physiological Conditions

Clinical, pathological, and molecular studies in AD patients and animal models have undoubtedly demonstrated the essential role of Aβ in the pathogenesis of AD. Indeed, the amyloidogenic hypothesis is one of the oldest theoretical approaches, being still broadly accepted today. According to general knowledge, Aβ is the result of the pathological cleavage of the amyloid precursor protein (APP) under β-secretase activity [28]. The detailed Aβ monomer formation process has been reviewed in one of the authors’ previous works [29]. From the pathological point of view, the abnormal Aβ monomer aggregation leads first to oligomers formation and, subsequently, to larger structures known as amyloid plaques. Recent research has demonstrated that oligomers, although initially thought to be harmless, have toxic effects at the neuronal level [30]. It is thought that Aβ oligomers are even more toxic than stable senile plaques, altering the normal synaptic transmission or axonal transport [31].

**Figure 1:**
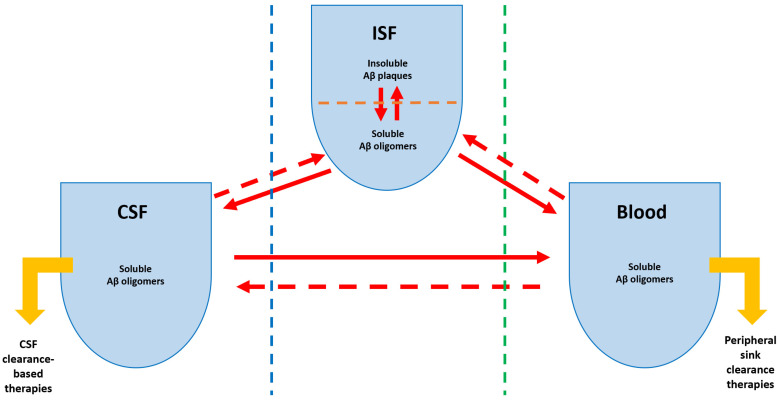
The schematic representation of the “three-sink therapeutic strategy” with the focus on amyloid-beta (Aβ) dynamics (modified from the work by Menendez-Gonzalez et al. [33]). The green line represents the blood−brain barrier; the blue line represents the brain−cerebrospinal fluid (CSF) barrier; the full red arrows indicate the clearance/efflux direction of Aβ; the dotted red arrows indicate the influx direction of Aβ; the yellow arrows indicate therapeutic strategies.

Aβ oligomers’ dynamics among different biologically significant compartments is an essential aspect from the therapeutic point of view. In a simplified functional anatomy classification, three biological compartments have great importance for AD evolution, diagnostic and, more recently, with potential therapeutic impact: the circulatory system (defined as the “peripheral sink”), the ventricular and subarachnoid compartments (known as the “CSF sink”), and the brain interstitial fluid (defined as the “central sink”). Aβ oligomers are soluble polypeptides and able to pass via the main limiting natural borders of these three distinct compartments. The blood−brain barrier (BBB), the blood−CSF barrier, and the CSF−brain barrier are the most relevant structures protecting the CNS, selectively controlling solute dynamics between the three compartments [32]. Several distinct mechanisms are involved in the bidirectional passage of Aβ between the CNS, blood, and CSF, as Figure 1 highlights.

At the CNS level, more precisely at the ISF level, Aβ can be found in two distinct forms, with different pathological and potential therapeutic impacts. On the one hand, Aβ aggregations can form stable pathological plaques, the essential hallmark of AD. Although the existence of these plaques was demonstrated several decades ago, recent research suggested that Aβ conglomerates are not pathognomonic for AD and might be found also in healthy controls, but with significant structural differences [34]. On the other hand, of greater interest is the soluble Aβ fraction located at the ISF level, as it can be more easily mobilized and was demonstrated to have higher toxicity for the brain parenchyma [35]. Moreover, when referring to interventional therapies aiming to decrease Aβ load from the CNS compartment, the soluble fraction is the principal target, as it can be easily removed and/or mobilized towards other biological systems (such as the CSF or the peripheral circulatory system).

Another limitation for future Aβ-directed therapies is the insufficient knowledge related to the mechanisms involved in Aβ transport via the BBB and other CNS barriers. The main Aβ clearance pathway from the CNS remains the BBB and involves several molecules. The BBB is a highly selective cellular complex that limits, in physiological conditions, the passage of neurotoxic substances and immune cells from the peripheral circulation to the brain parenchyma [36]. The BBB is essential for the maintenance of brain homeostasis, its leakage being linked to many neurological disorders, including AD [37]. In a healthy brain, brain protein clearance to the peripheral circulatory system is mediated by several BBB membrane transporters. One of the most studied carriers of the specialized transport system is P-glycoprotein 1 (P-gp), known also as ATP Binding Cassette Subfamily B Member 1 (ABCB1), the main transporter in the ABC transporters family [38]. ABCA1, another member of the same transporters’ family, initially considered to be involved in the protection against atherosclerosis [39], seems to play an important role in cerebral Aβ efflux and subsequently in AD pathogenesis [40]. Two members of the low-density lipoprotein (LDL)-receptor protein family, namely LDL receptor-related protein 1 (LRP1) and LRP2, were also demonstrated to be involved in Aβ clearance, at different levels, including the BBB, but also the choroid plexus [41]. α2-macroglobulin (α2M) is another molecule associated with the mediation of Aβ clearance and other pro-amyloidogenic factors that promote AD evolution [42]. Finally, the insulin-degrading enzyme (IDE) seems to gain significant ground in the understanding of Aβ dynamics. IDE is thought to inhibit Aβ fibrillogenesis, with studies suggesting specific gene polymorphisms to be linked to an increased risk of late-onset AD (LOAD) [43].

The influx transport of Aβ should also be mentioned, as it is heavily altered in pathological conditions such as AD. The main effector involved in the facilitation of Aβ reentry in the CNS is the advanced glycosylation end-product-specific receptor (RAGE) [44]. This transmembrane receptor is considered to establish a positive feedback cycle in pathological conditions (including AD), leading to subsequent chronic inflammation via pro-inflammatory gene activation [45].

Several other CNS protein clearance pathways were described, direct end-product degradation being a relevant one. Protein degradation can occur at both the intra- and extracellular levels via different mechanisms. Other recent works have already reviewed the role of intracellular systems including the ubiquitin−proteasome [46], autophagy−lysosome [47], and endosome–lysosome pathways [48] in physiological conditions and in neurological disorders, when they are altered. Similarly, extracellular degradation occurs via protease action and microglia or astrocyte phagocytosis, being significantly reduced in pathological conditions such as AD [49]. Finally, another two incompletely understand and underexplored clearance pathways gain increasing importance as they might become therapeutic targets in the near future. The perivascular drainage system consists of mostly passive diffusion of ISF solutes along the capillary and arterial brain vessels [50]. Although much reduced compared to the BBB clearance, this system may compensate in case of the failure of other cerebral clearance pathways in pathological conditions, or it can be directly involved in vascular pathology such as amyloid angiopathy [51]. Additionally, the “glymphatic” system, the most recently described para-vascular drainage system [52], facilitates solutes clearance from the ISF via aquaporin 4 (AQP4) water channels and other insufficient explored mechanisms. In normal conditions, only some roles of the “glymphatic” system are described, such as waste clearance predominant during sleep [53]; however, emerging functions are expected to be discovered.

ISF Aβ can also flow to the peripheral circulatory system indirectly via the CSF. The main structural border is represented by the CSF−brain barrier, another unique structure in the human body that separates the cerebral parenchyma from the CSF, regulating fluidic crosstalk between the two compartments.

After exiting the cerebral reservoir, Aβ at the CSF level is directed toward the “peripheral sink” where the final steps of catabolism take place. Via several absorption mechanisms, Aβ from the CSF flows primarily to the peripheral circulatory system and, secondarily, in much lower concentrations, to the lymphatic vessels. CSF filtration occurs at two distinct places at the CNS level, in the arachnoid villi and at the brain−CSF barrier (BCSFB). The arachnoid villi, located along the dural sinuses (mostly along the superior sagittal sinus), are microscopic herniations of the arachnoid membrane through the dura mater and function like valves that allow unidirectional CSF flow [54]. The BCSFB, contrarily, is like the BBB, a specialized structure located at the choroid plexus that separates in a highly controlled and selective manner bidirectional solute transport between the ISF and blood [55]. Initially considered to be the source of CSF, recent research demonstrated that choroid plexuses have also CSF absorption capacity, filtrating small molecules with clinical impact, including soluble Aβ [56].

### 2.2. The Pathological Dynamics of Aβ in LOAD

The Aβ clearance via the abovementioned specialized structures has become of increasing interest, given their role in LOAD, a pathological condition with significant impact. The pathogenesis of AD is represented by the excessive production of Aβ in only a minority of patients, who are considered to suffer from the inherited, early-onset form of AD [57]. In the rest of almost 95% of AD patients, the main disease mechanism is the impaired Aβ clearance in the context of normal (or slightly exaggerated) production [58]. There is a significant reduction in most of the presented clearance pathways in AD patients. For example, in the case of BBB alterations, both a reduced efflux and an increased influx of solutes (including Aβ) were observed. The BBB is disrupted in AD via a series of complementary mechanisms such as sustained neuroinflammation, oxidative stress, and dysregulated autoimmunity, resulting in the downregulation of carriers such as P-gp and LRP1, concomitantly with the upregulation of RAGE and other influx transporters [59]. Furthermore, the catabolism of misfolded proteins is also altered in AD, the severe modifications in microglia and astrocyte activity impairing intracellular degradation [60]. Autophagy and endolysosomal systems are also disturbed starting from the early phase of AD, probably as a result of genetic mutations of *APOE4*, *BIN1*, or *PICALM*, favoring Aβ build-up [61]. Despite being considered a secondary Aβ clearance pathway, perivascular drainage is also altered in AD and cannot compensate for the dysfunction of the other clearance systems. Still possessing many unknowns for researchers, recent studies pointed toward the pathological enlargement of the perivascular spaces as an early marker for neurodegeneration [62]. Age-related factors including atherosclerosis and elastin dysfunction negatively modulate the vascular environment, thus reducing the physiological elasticity of the arterioles which subsequently compromises the role of perivascular spaces in brain waste drainage. A similar mechanism is also suspected to lead to the impairment of the “glymphatic” system by blocking the filtration of solutes and altering the APQ4 channel function. Finally, dysfunctional Aβ dynamics might also be encountered at the peripheral level, the importance of peripheral dysfunctions in CNS neurodegeneration still being completely elucidated.

## 3. The “CSF-Sink Therapeutic Strategy”

**Figure 2:**
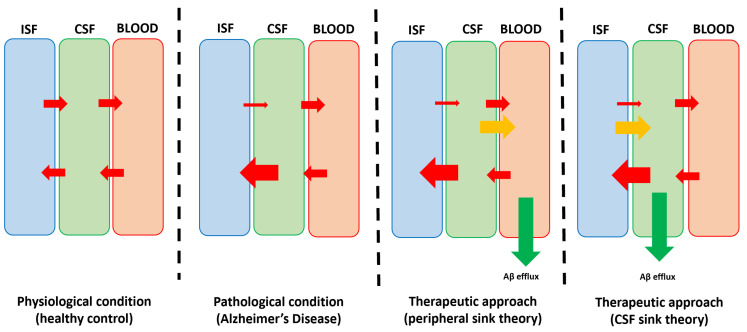
Aβ dynamics in physiological, pathological, and interventional conditions (peripheral sink therapeutic strategy and CSF-sink therapeutic strategy). The red arrows indicate Aβ influx/efflux. In AD, the decreased Aβ efflux is concomitant with the increased Aβ influx. In the peripheral sink therapeutic strategy, the increased Aβ elimination from the peripheral circulation (green arrow) stimulates the Aβ efflux from the CSF (yellow arrow), but not from the brain. In the CSF-sink therapeutic strategy, the increased Aβ elimination from the CSF (green arrow) stimulates the Aβ efflux from the interstitial fluid (ISF; yellow arrow).

Reviewing all known Aβ clearance pathways, the common point of cerebral waste drainage is the CSF, an intermediary but essential compartment for many protein compounds, including misfolded Aβ physiological drainage. Despite the alterations of Aβ cerebral efflux in pathological conditions (such as AD), significant amounts of solute reach the CSF and are subsequently directed to the peripheral circulation. Starting from these theoretical findings, the CSF is considered to be an essential therapeutic target. Via specific interventions designed to change the CSF biochemical characteristics (reduction of certain solutes), the reduction in CSF Aβ concentration might lead to alterations of Aβ at the cerebral level. As mentioned in previous works [33,63], the “CSF-sink therapeutic strategy” is based on the dynamic equilibrium of solutes between three distinct compartments, i.e., ISF, CSF, and blood. Lowering the solutes level in one of these “sinks” leads, hypothetically, to the reduction of that specific solute in the brain parenchyma which represents the main therapeutic goal. Figure 2 summarized the theoretical concepts on which future therapies might be based.

The CSF compartment is already a therapeutic target in a different class of neurological diseases, the pathological enlargement of the CSF ventricular system, namely hydrocephalus. Hydrocephalus etiology and subtypes are not be discussed here, as they surpass the scope of this review; however, the surgical therapeutic approaches are relevant for the development of future interventional techniques which might be effective also in neurodegenerative diseases. The current gold standard in treating obstructive and non-obstructive hydrocephalus is the diversion of CSF from the lateral ventricles to a peripheral compartment, usually the peritoneum [64]. Having already a vast experience with CSF-shunting devices which implies both advantages and possible complications, the know-how could be easily translated into the field of AD. In order to make devices known as “CSF clearance pumps” viable for daily clinical use, some mandatory improvements are urgently needed. Firstly, the optimization of the device design is essential, as currently available shunts are still big, uncomfortable equipment associated with rare, but unpleasant side effects/complications such as infections or hemorrhages [65]. Secondly, the filtration parameters (including capacity and sensitivity) should be improved. While for hydrocephalus treatment, the main objective is to decrease the surplus of CSF from the ventricular system; in neurodegenerative disease, one of the potentially curative approaches is to diminish the misfolded protein (hyperphosphorylated Tau protein, pathological Aβ aggregates) levels from the CSF which subsequently reduces the cerebral load. In order to obtain this, several methods should be combined, including mechanical (tiny pores and passive filtration) and immunological (specific antibodies) for increased success rates. With many therapeutical trials already failing (see below in Section 4), this more direct and minimally invasive method could bring certain advantages for AD patients, if not in a curative manner, at least from a prophylactic point of view to delay AD onset and slower disease progression.

## 4. AD Therapeutic Approaches Focused on Aβ Clearance

Although currently without effective long-term treatment, AD is one of the most studied diseases in human medicine, with a huge number of clinical trials conducted so far. An extensive search in the main online databases (PubMed, Cochrane, and Google Scholar) reveals that numerous and heterogeneous approaches were tried throughout the last decades, unfortunately with negative results. Table 1 summarizes the main therapeutic directions followed up to the present.

When referring to the Aβ equilibrium, two distinct processes can be modulated in order to reduce the Aβ cerebral load: diminishing the production and increasing the elimination. Earlier research focused more on reducing Aβ production, the amyloidogenic pathway being the starting point for developing anti-amyloid drugs. An interesting attempt was to target the β-secretase, beta-site amyloid precursor protein-cleaving enzyme (BACE) inhibitors, especially BACE1 inhibitors, being among the first studied molecules in AD trials [66]. Despite the initial hype, trials failed, as besides their inefficiency in reducing Aβ cerebral load, their administration was also associated with increased brain atrophy and weight loss [67]. Moreover, a severe elevation of liver enzymes was detected as a side effect of altered immunity in patients receiving a specific medication (Atabecestat) [68]. One explanation for treatment failure is the AD mechanism itself, based on altered Aβ clearance in a far greater proportion compared to pathological Aβ production. Thus, despite lowering Aβ production, Aβ continues to accumulate at the brain level, as degradation and efflux to CSF are impaired. According to recent opinions, BACE still remains a valuable therapeutic target, and the development of novel molecules with fewer side effects is essential at least as a possible adjuvant therapy in the near future [69].

RAGE inhibitors, currently under scrutiny, are another class of medication that targets a relevant molecule involved in the Aβ dynamics. RAGE, the main influx transporter at the BBB level, is overexpressed in AD, leading to disturbances of Aβ levels between the cerebral sink and the CSF sink. In this context, inhibiting RAGE should decrease Aβ influx, leading to subsequent lower accumulation in the brain. Moreover, RAGE is related to the chronic inflammatory process, linking Aβ to microglial pathological activation [70]. Silencing RAGE means reducing neuroinflammation, one of the main components in neurodegenerative disorders such as AD [71]. Up to the present, Azeliragon has been the only drug that manages to achieve stage III clinical trials, but research was stopped earlier due to the lack of end-points meet.

As therapeutic tactics oriented toward reducing cerebral Aβ production and influx have not achieved the endpoints of the clinical trials and failed to demonstrate efficacy, different approaches focusing on the enhancement of Aβ drainage were studied. Considering the Aβ dynamics among biological compartments, an easily applicable strategy was to evacuate Aβ from the peripheral circulation and to track if changes in Aβ concentration at the central level occur. Two distinct immune-based approaches are available, active and passive immunotherapy which neutralizes Aβ and subsequently favors its elimination. Regarding active anti-Aβ immunotherapy, earlier results were almost catastrophic. Trials on the first AD vaccine (AN-1792) were prematurely stopped because of severe adverse effects (meningoencephalitis) and higher rates of death compared to placebo [72]. However, with consistent evidence of Aβ plaque load reduction after immunization [73], new attempts are currently made, with definite conclusions to be drawn in the next few years. Passive anti-Aβ immunotherapy, on the other hand, has recorded much more optimistic results. With more than three drugs currently in phase III clinical trials, monoclonal antibodies show a higher safety administration rate, with fewer adverse reactions. Indeed, starting from June 2021, Aducanumab is the first FDA-approved anti-Aβ antibody available for AD patients [74]. This fast-forward approval has generated at the same time a fierce debate in the research community, as its impact on cognition and the functional decline was not completely demonstrated [75].

Learning from past failed attempts, future therapies should approach AD in a holistic manner. The combination of more than one therapy means focusing on several disease mechanisms with a cumulative effect on the Aβ cerebral level reduction. In this context, running research is conducted on neprilysin delivery at the CNS and peripheral levels, with the final aim to enhance Aβ degradation [76]. Physical−mechanical strategies based on filtration, such as plasmapheresis and dialysis, could also have a significant clinical impact, as preliminary results show an association to slower cognitive and functional decline in treated AD patients compared to placebo [77]. A novel, very promising strategy can be the use of a combined Aβ filtration from different biological compartments (including CSF) and Aβ neutralization via the use of specific antibodies, an underexplored therapeutic direction that is presented from our perspective and research experience below.

## 5. Implantable Devices for AD CSF Therapy—System Design and Application

**Figure 3:**
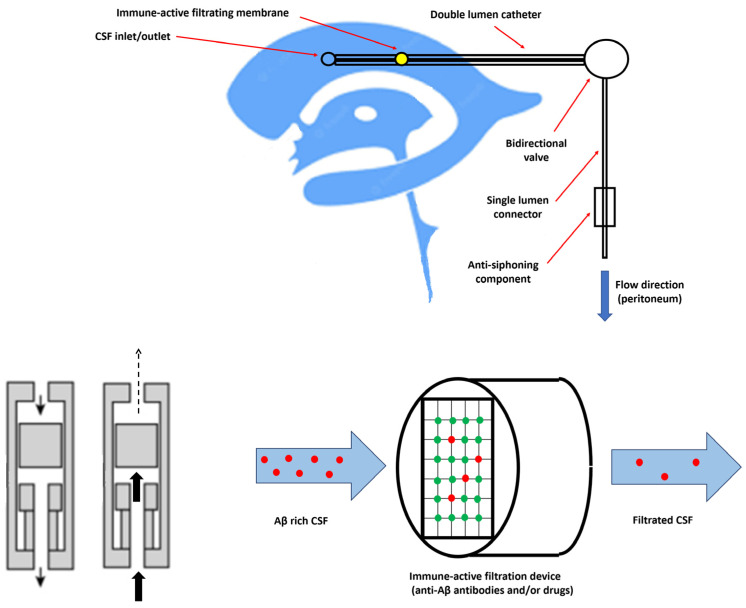
The proposed design of a CSF immune filtration device. A siphon-resistant valve impedes antigravitational flow, when the patient changes body position. Detailed view of the immune-active filtrating membrane: specific anti-Aβ drugs and/or antibodies (green dots) bind Aβ (red dots) from the CSF.

As Aβ clearance from the “peripheral sink” does not achieve the expected clinical results [78], focusing on CSF clearance is the next logical step in the search for an efficient anti-amyloid therapy. In this regard, the authors proposed a special device capable of CSF immune filtration, subsequently reducing the Aβ at the CSF level. According to the schematic representation in Figure 3, the equipment should contain some mandatory components and meet certain parameters in order to complete its therapeutic role in AD patients.

A double lumen catheter (with both the input and the output) is preferred, as it allows the simultaneous draining and reinfusion of CSF to the ventricular system. Moreover, the inlet lumen could be used for the delivery of therapeutic substances directly at the CSF level. The structural characteristics (size, length, and gauge) of the catheter are equally important, as the device should be suitable for both the ventricular and intrathecal (lumbar region) placement. The double lumen catheter should include a bi-directional valve designed to control influx and efflux rates. The use of a variable pressure valve is recommended, as corrections could be needed during the Aβ clearance-based treatment. Inside the core of the device, an immune-active membrane should be mounted. Containing specific antibodies against targeted molecules (for example anti-Aβ antibodies), this tiny-pore membrane should retain the soluble Aβ mono- and oligomers, permitting free flow for other solutes. Moreover, instead of specific antibodies, other highly efficient anti-Aβ drugs such as neprilysin can be attached to the filtrating grid, destroying the excess Aβ from the CSF. Finally, an external pump ensures continuous CSF flow through the tubing, in order to prevent excessive CSF efflux. One severe complication that should be avoided is the iatrogenic CSF leakage syndrome caused by excessive drainage which is not compensated by reperfusion. Headache, neck stiffness, vomiting, dizziness, and tinnitus are the main symptoms that should urgently suggest CSF overdrainage related to dysfunctions of the implanted device [79].

The most important feature when designing a new implantable device is the biocompatibility of the used materials. Biomedical interface materials used nowadays still rely heavily on older “commodity polymers” developed over 40 years ago and known for their associated potential adverse reactions such as infections and thrombosis. The sensitive cerebral and/or spinal tissues require even more human-friendly components, especially for long-term implanted devices. Nanotechnology and tissue engineering are two essential directions where advances should be made in order to create biocompatible systems with minimized side effects.

Regarding the optimal functioning parameters, there are no clear statements within international guidelines, and individual tailoring is frequently necessary. The clearance rate, an important parameter measured in a drained volume per time unit (minute, hour, or day), is dependent on the physiological CSF production and retained clearance. CSF production is the only constant among the hydrodynamic parameters, in humans having a mean value of 0.35 mL/min, almost 500 mL daily [80]. The variability of other hydrokinetic forces is dependent on many external factors, the gravitational attraction being one of the most significant factors dictating the filtration rate. Related to the gravity force, the “siphon effect” is another potential complication that should be considered. In order to counterattack this phenomenon (and to prevent potential complications such as subdural hematoma), the use of siphon-resistant devices is recommended. Several mechanical solutions were found, with the employment of membranous or gravitational devices reducing the amount of siphoning [81]. Finally, real-time parameter measurements are the most desired in order to apply a personalized treatment. Intracranial pressure and CSF flow changings should be promptly detected by incorporated biosensors or other minimally-invasive monitoring devices; however, much innovation is needed, as currently available systems have major limitations.

With research currently ongoing and still many unknowns to be explored, a last topic of interest remains the duration of treatment. As CSF-shunting devices are not designed for permanent use, they are more suitable for bridging or preventive therapy. When considering AD prevention, several questions arise. The moment of implantation, the profile of an appropriate patient, the treatment duration, and the filtration ratio are only some of the unanswered issues that must be critically established before applying this treatment on a large scale. As Aβ pathological formation precedes dementia symptoms by years, CSF drainage in pre-dementia and mild cognitive impairment patients could delay AD onset. Future research will demonstrate whether CSF-shunting devices are appropriate methods of AD prevention.

## 6. Conclusions

With incidence and prevalence expected to increase in the next years, AD will remain one of the most commonly encountered neurological disorders worldwide. Despite intense research, there is no curative treatment available, and the existing therapies bring only limited benefits to AD patient. In this context, both a better understanding of the disease and the development of therapeutic strategies are essential.

Among the multiple etiopathogenic hypotheses that try to explain AD onset, the amyloidogenic hypothesis still has some valuable data to offer in the context of innovations regarding therapeutic methods based on Aβ clearance. Formulated on the classical theory, a new concept called the “three-sink therapeutic strategy” focuses on the dynamic equilibrium of Aβ among the most important biological compartments of the human body. According to this principle, modifications in the CSF or peripheral Aβ concentrations could lead to changes in the cerebral Aβ level.

With the possibility of applying therapeutic devices at the “CSF-sink” level, managing Aβ levels in the CSF should have a significant impact on cerebral Aβ and disease control. One key feature for successful AD management relies on coupling several Aβ clearance methods within the same therapeutic device. In this context, our preliminary system design offers a good starting point for the development of more patient-friendly CSF clearance devices that need mandatory further developments and testing.

## Figures and Tables

**Table 1:** The diversity of Alzheimer’s disease therapeutic options learned from failure.

Proposed Therapy	Studied Substance/Method	Most Significant Clinical Trials	Reasons for Failure/Current Status
BACE (mostly BACE1) inhibitors	Verubecestat	EPOCH	Increased mortality Cognitive worsening Rapid hippocampal volume reduction
	Lanabecestat	AMARANTH DAYBREAK-ALZ	Primary end-points not met
	Atabecestat	EARLY	Liver enzymes elevation
RAGE inhibitors	Azeliragon (PF-04494700)	STEADFAST	End-points not met
Active anti-Aβ immunotherapy	AN-1792	NCT00021723	Meningoencephalitis development Six deaths
	CAD106	NCT01097096	Trial ongoing
	ABvac40	NCT03461276	Trial ongoing
Passive anti-Aβ immunotherapy	Solanezumab	EXPEDITION (1, 2, and 3)	End-points missed Safety concerns: ARIA-E and ARIA-H
	Gantenerumab	SCarlet RoAD Marguerite RoAD	Trials ongoing
	Lecanemab	NCT03887455	Trials ongoing
	Aducanumab	ENGAGE EMERGE	First FDA-approved human recombinant Aβ antibody
Aβ cleavage	Neprilysin	Intracerebral and peripheral delivery	Trials ongoing
Peripheral-sink Aβ clearance	Plasmapheresis	AMBAR	Trials ongoing
	Peritoneal dialysis	-	Trials ongoing
CSF-sink Aβ clearance	Intrathecal drug delivery systems	-	Trials ongoing

Abbreviations in Table 1: BACE, beta-site amyloid precursor protein cleaving enzyme; RAGE, advanced glycosylation end-product-specific receptor; FDA, Food and Drug Administration; CSF, cerebrospinal fluid; ARIA-E/H, amyloid-related imaging abnormalities edema/hemorrhages.

## Data Availability

All data and materials supporting the results of the present study are available in the published article.
